# Intravenous infusion of the exosomes derived from human umbilical cord mesenchymal stem cells enhance neurological recovery after traumatic brain injury via suppressing the NF-κB pathway

**DOI:** 10.1515/biol-2022-0022

**Published:** 2022-03-17

**Authors:** Zhen-Wen Zhang, Pan Wei, Gui-Jun Zhang, Jing-Xing Yan, Sai Zhang, Jin Liang, Xiao-Li Wang

**Affiliations:** Department of Encephalopathy, Affiliated Hospital of Gansu University of Chinese Medicine, Lanzhou 730000, Gansu, China; Tianjin Key Laboratory of Neurotrauma Repair, Pingjin Hospital Brain Center, Characteristic Medical Center of PAPF, Tianjin 300162, China; College of Integrated Traditional Chinese and Western Medicine, Gansu University of Chinese Medicine, Lanzhou 730000, Gansu, China; Department of Neurosurgery, The First People’s Hospital of Long Quan Yi District, Cheng Du 610000, Si Chuan, China; Department of Neurosurgery, West China Medical School, West China Hospital, Sichuan University, Chengdu 610041, Sichuan, China

**Keywords:** exosomes, human umbilical cord mesenchymal stem cells, neurological recovery, NF-κB, traumatic brain injury

## Abstract

Traumatic brain injury (TBI) is a predominant cause of death and permanent disability globally. In recent years, much emphasis has been laid on treatments for TBI. Increasing evidence suggests that human umbilical cord mesenchymal stem cells (HUCMSCs) can improve neurological repair after TBI. However, the clinical use of HUCMSCs transplantation in TBI has been limited by immunological rejection, ethical issues, and the risk of tumorigenicity. Many studies have shown that HUCMSCs-derived exosomes may be an alternative approach for HUCMSCs transplantation. We hypothesized that exosomes derived from HUCMSCs could inhibit apoptosis after TBI, reduce neuroinflammation, and promote neurogenesis. A rat model of TBI was established to investigate the efficiency of neurological recovery with exosome therapy. We found that exosomes derived from HUCMSCs significantly ameliorated sensorimotor function and spatial learning in rats after TBI. Moreover, HUCMSCs-derived exosomes significantly reduced proinflammatory cytokine expression by suppressing the NF-κB signaling pathway. Furthermore, we found that HUCMSC-derived exosomes inhibited neuronal apoptosis, reduced inflammation, and promoted neuron regeneration in the injured cortex of rats after TBI. These results indicate that HUCMSCs-derived exosomes may be a promising therapeutic strategy for TBI.

## 1 Introduction

The pathological hallmarks of traumatic brain injury (TBI) include neuron loss, axonal destruction, and demyelination [1], accounting for poor patient neurological recovery. Although unprecedented progress has been made in recent years, the treatment of TBI remains highly challenging. TBI can lead to physical disability, neurobehavioral dysfunction, and cognitive impairment [2,3,4,5,6,7,8]. Currently, the mechanisms underlying the pathological changes in TBI remain largely understudied. It is well established that neuroinflammation plays a crucial role in secondary TBI [9] involving many cell types, especially microglia and astrocytes involved in inflammatory reactions [10,11]. Astrocytes have many important physiological functions, such as providing trophic support, maintaining homeostasis, modulating synapses, and maintaining the blood-brain barrier. Serious brain insult results in reactive astrocytosis, which can both sustain and impede the recovery of the central nervous system [12,13,14]. Recent studies have suggested that astrocytes can migrate to neurotoxic A1 or neuroprotective A2 phenotypes via a stimulus-specific manner, and A1 astrocytes activation led to neuronal cell death. In contrast, A2 astrocytes activation led to a protective effect against inflammation and protected neurons by upregulating many neurotrophic factors [15].

Mesenchymal stem cells (MSCs) treatment has been subjected to great interest in recent years since they can promote restoration of neurological function after treatment [16,17]. However, MSCs treatment has been associated with some disadvantages in the clinical setting. Previous studies have shown that MSCs treatment may increase the tumorigenic risk [18,19], and only a small portion of the transplanted MSCs survive and differentiate into neurons in the damaged brain tissues [20]. Although it is widely acknowledged that MSCs can repair brain tissue damage [21], little is known about the underlying neuroprotective mechanisms. Several reports have shown that the repair and restorative functions of MSCs involve paracrine mechanisms, not a transdifferentiation [22].

Exosomes are lipid bilayer membrane vesicles released from various cell types, ranging from 40 to 160 nm in diameter [23]. Exosomes are abundant in endosome-derived components, including lipids, proteins, mRNAs, and microRNAs (miRNAs) [23]. An increasing body of evidence suggests that MSCs-derived exosomes exert neuroprotective effects [24] by improving neurite remodeling and neurofunctional recovery after TBI [25] and stroke [26]. Studies have shown that activation of the NF-κB signaling pathway results in the synthesis of proinflammatory cytokines such as TNF-α and IL-6 [27]. It is unclear whether the intravenous infusion of exosomes derived from human umbilical cord mesenchymal stem cells (HUCMSCs) reduces inflammation and promotes functional recovery through the NF-κB signaling pathway. This study assessed the effects of HUCMSCs-derived exosomes on functional recovery and explored the potential mechanisms underlying their neuroprotective effects after TBI.

## 2 Materials and methods

### 2.1 Isolation, culture, and identification of HUCMSCs

HUCMSCs were isolated from healthy newborn’s umbilical cords from our hospital’s maternity department according to an established method [28,29]. The cells were then cultured in DMEM/F12 medium (Gibco, Grand Island, NY, USA). (containing volume fraction 10% fetal bovine serum (FBS; MRC Biotechnology Co., Ltd, Jiangsu, China), 25 mmol/L glutamine (Solarbio Science & Technology Co., Ltd, Beijing, China), 100 U/mL penicillin (Sigma-Aldrich, St. Louis, MO, USA), 100 mg/L streptomycin (Sigma-Aldrich, St. Louis, MO, USA) in a 5% CO_2_ incubator at 37°C. The morphology of HUCMSCs was observed by inverted phase-contrast microscopy.

Immunofluorescence was performed to identify the immunological phenotype of HUCMSCs. Cell samples were incubated with rabbit polyclonal antibody against CD90 (1:300, Abcam, Cambridge, UK) and mouse polyclonal antibody against CD105 (1:400, Abcam, Cambridge, UK) at 4°C overnight. A fluorescence microscope (Leica TCS SP5, Germany) was used for observation and photographing.

HUCMSCs were identified by flow cytometry, as previously described [28,29]. Briefly, the cell suspension to be tested was separated into 8 tubes (100 μL per tube). These cells were incubated with CD90, CD105, CD73, CD116, CD19, CD45, and HLA-DR antibodies (Abcam Cambridge, UK) for 30 min at 4°C according to the instructions of the flow cytometry kit. The positivity rate of various antigens was determined by a Cytomics FC500 flow cytometer (BD Biosciences, San Jose, CA).


**Informed consent:** Informed consent has been obtained from all individuals included in this study.
**Ethical approval:** The research related to human use has been complied with all the relevant national regulations, institutional policies and in accordance with the tenets of the Helsinki Declaration, and has been approved by the authors’ institutional review board or equivalent committee.

### 2.2 Isolation and identification of exosomes derived from HUCMSCs

Exosomes were isolated from HUCMSCs using methods previously described [30]. Briefly, the medium harvested from HUCMSCs was centrifuged at 500×*g* for 30 min, at 3,000×*g* for 30 min at 4°C and at 5,000×*g* for 30 min to obtain the supernatant. The supernatant was placed in a 30% heavy water sucrose pad in an ultra-detached tube and subjected to ultracentrifugation at 120,000×*g* for 70 min at 4°C. The heavy water sucrose cushion was washed twice with PBS at 120,000×*g* for 70 min. The precipitate used in the DMEM/F12 medium was resuspended in the tube wall and filtered through a 0.22 μm sterile filter for subsequent experiments.

Exosomes were identified by transmission electron microscopy (TEM; HT7700, Hitachi, Japan) [31]. The sample was then photographed using a transmission electron microscope. Western blot was performed to quantify the expression of exosome surface markers (CD9 and CD63) according to established methods [32].

### 2.3 TBI model and exosome therapy

Male Sprague-Dawley (SD) rats (weight 250–300 g) were obtained from the Military Academy of Medical Sciences of the People’s Liberation Arm. All rats were housed under standard environmental conditions: 12 h light/dark cycle, temperature (22 ± 1°C), wire-top-type cages, three rats per cage, and free access to water and food. All experiments were carried out between 9:00 and 12:00 am.

The rats were subjected to Cortical Contusion Impact Injury by using an electric cortical contusion impactor device (eCCI-6.3; Custom Design & Fabrication) according to established methods [33]. Briefly, rats were anesthetized by intraperitoneal injection of pentobarbital sodium (50 mg/kg). A bench drill was used to puncture a circular orbital window of diameter 5 mm (2 mm on the posterior side of the coronal suture and 1 mm on the right side of the sagittal suture). The rat was then moved to an electronic eCCI console, the hammer position was adjusted to align with the open bone window and perpendicular to the cortex, and the striking parameters were set as follows: speed 5 m/s, depth 2 mm, hammer dwell time 120 ms. After the injury, hemostatic methods were quickly applied. Immediately after the injury, the small animal ventilator was used to provide breathing assistance, and if necessary, chest compressions were given. The scalp incision was sutured after normal breathing was resumed.

Forty-five rats were randomly divided into three groups: Sham group (*n* = 15), TBI group (treated with PBS, *n* = 15), and Exo group (treated with exosome, *n* = 15). The Sham group rats underwent a bone drill without moderate severity brain injury. The TBI group was subjected to moderate severity brain injury and then injected with intravenous PBS over 5 min via the tail vein, starting 1 day after injury. The Exo group was subjected to moderate severity brain injury and then intravenously injected with exosomes derived from HUCMSCs (100 μg total protein of exosome precipitate in 0.5 mL of PBS per rat) over 5 min via the tail vein, starting 1 day after injury.


**Ethical approval:** The research related to animal use has been complied with all the relevant national regulations and institutional policies for the care and use of animals, and was approved by the Animal Care and Use Committee of the People’s Armed Police Logistics Institute (PAP) (Approval No. 2019-0019.5) and PAP Research Animal Ethics Committee (ethical approval reference number 36569/62).

### 2.4 Behavioral testing

#### 2.4.1 Modified neurological severity scores (mNSS)

In the TBI and Exo groups, mNSS were used to evaluate neurological function 3 h after modeling (*n* = 15 for each group), and rats with an mNSS score <10 were excluded. Fifteen rats were randomly selected from each group, and the mNSS scores were graded at 7, 14, 21, and 28 days after modeling. The mNSS assessment consists of an exercise test (lifting test and abnormal activity), sensory test (visual, tactile, and proprioception), balance beam test, reflex activity, and abnormal movement. The mNSS score is usually graded on a scale of 0–18, the normal score is 0, and the maximum deficit score is 18, the lower the mNSS score, the better the neurological function [34].

#### 2.4.2 Morris water maze (MWM) test

To assess the possible effects of MSCs on the cognitive function in rats after TBI, spatial learning and memory in rats were measured by the MWM test (*n* = 15 for each group) [29]. During the learning process, the ability of rats to find the submerged platform was trained for 21–26 days after TBI, and the time required for the rats to enter the water to find the platform (the escape latency time) was recorded. The platform was removed on the 28th day after the injury, and the spatial learning and memory ability of the rats were evaluated by measuring the latency time spent in the target quadrant and the number of platform crossings.

#### 2.4.3 Long-term potentiation (LTP) measurement

After TBI, SD rats will develop memory deficits. To demonstrate the improvement in memory deficits in TBI rats, LTP was measured to study synaptic transmission [35,36,37]. The electrophysiological measurements were carried out at 28 days after TBI according to a method previously described [29]. Briefly, the rats were fixed to a stereotactic instrument after anesthesia (*n* = 15 for each group). The skull was exposed, then a small hole was formed on the opposite side of the injury to achieve vertical penetration by the stimulation and recording electrodes. According to the rat brain atlas, the stimulating electrodes were placed in the area at the following corresponding coordinates: AP –6.8 mm, ML 4.5 mm, and DV –3.5 mm. The recording electrode is placed in the area of the corresponding coordinates (−3.5 mm on the AP, ML 3.4 mm, and DV −3.5 mm). A stable baseline for at least 20 min was needed before each stimulus was applied during each electrophysiological recording experiment. LTP was initiated by high-frequency stimulation (HFS) consisting of 4 pulses in a series of 50 pulses delivered at 200 Hz with an intertrain spacing of 2 s. A computer program (RM6240BD; Chengdu, China) was used to analyze LTP by calculating excitatory postsynaptic potential (EPSP) and population peaks (PS).

### 2.5 Enzyme-linked immunosorbent assay (ELISA)

At 3 days after TBI, the expression of inflammatory factors such as Tumor necrosis factor-alpha (TNF-α), Interleukin (IL)-1β, and IL-6 was measured using ELISA kits (R&D Systems, Minneapolis, MN), according to the manufacturer’s instructions. After the tissue in the injured area was obtained, the sample was prepared using a grinder and an ultrasonic tissue homogenizer. A microplate reader is used to obtain the optical density (OD) value at 490 nm. Finally, the expression levels of the three inflammatory factors were measured according to the kit instructions.

### 2.6 Western blot analysis

Western blot was used to measure the expression of the NF-kB signaling pathway at 3 days after TBI. Following electrophoresis separation, proteins were transferred to the PVDF membrane. The membranes were then blocked and incubated with primary antibody, mouse anti-NF-κBP65 antibody (1:500; Cell Signaling Technology, Shanghai, China), and anti-β-actin antibody (1:2,000; Sigma) at 4°C overnight. Then, the membranes were incubated with the secondary antibody (1:1,000, Cell signaling) for 2 h at room temperature (RT). The protein bands were determined using an image analysis system.

### 2.7 TUNEL (terminal deoxynucleotidyl transferase dUTP nick end labeling) staining

Apoptosis analysis of the cerebral cortex was performed by TUNEL (*In Situ* Cell Death Detection Kit; Roche Diagnostics, Indianapolis, IN, USA) staining at 3 days after TBI [38]. Briefly, brain tissue sections were counterstained with DAPI for 15 min. After the sections were washed twice with PBS, the sections were then stained with the TUNEL reaction mixture for 60 min at a constant temperature of 37°C. Cell quantification was implemented by two observers blinded to the experiment using a fluorescence microscope at a magnification of 200×. The percentage of apoptotic cells was obtained using the formula [39]:

\text{Positive}\hspace{.25em}\text{number}\hspace{.25em}\text{of}\hspace{.25em}\text{cells/(Positive}\hspace{.25em}\text{cells}+\text{Negative}\hspace{.25em}\text{cells)}\times 100 \% .]

### 2.8 Immunofluorescent Staining

To assess the effect of exosome treatment on inflammatory response in the cerebral cortex after craniocerebral trauma, brain tissue sections were labeled with Iba1 (a microglia marker) at 7 days after TBI, CD68 (to identify activated macrophages/microglia), and glial fibrillary acidic protein (GFAP) (to identify reactive astrocytes) at 28 days after TBI. To assess the effect of exosome treatment on the cerebral cortex changes, brain tissue sections were labeled with Doublecortin (DCX, a marker of immature neurons) at 7 days after TBI and neuronal nuclear protein (NeuN, a marker of mature neurons) at 28 days after TBI.

The immunofluorescence assay was carried out according to previously described methods [2]. The slices were incubated with primary antibodies (Iba1, 1:500; CD68, 1:1,000; GFAP, 1:400; DCX, 1:100; and NeuN, 1:200) (Abcam, Cambridge, UK) at 4°C overnight. A fluorescence microscopy (Leica TCS SP5, Germany) was used for observation and photographing. Cell counting was performed according to established methods [25,40].

### 2.9 Statistical analysis

Data were expressed as mean value ± standard deviation (SD), and the differences were analyzed using One-way ANOVA and student *t*-tests with GraphPad Prism 6 software (GraphPad Software, Inc., San Diego, CA, USA). A *P*-value <0.05 was statistically significant.

## 3 Results

### 3.1 Culture of HUCMSCs and identification of surface markers

**Figure 1:**
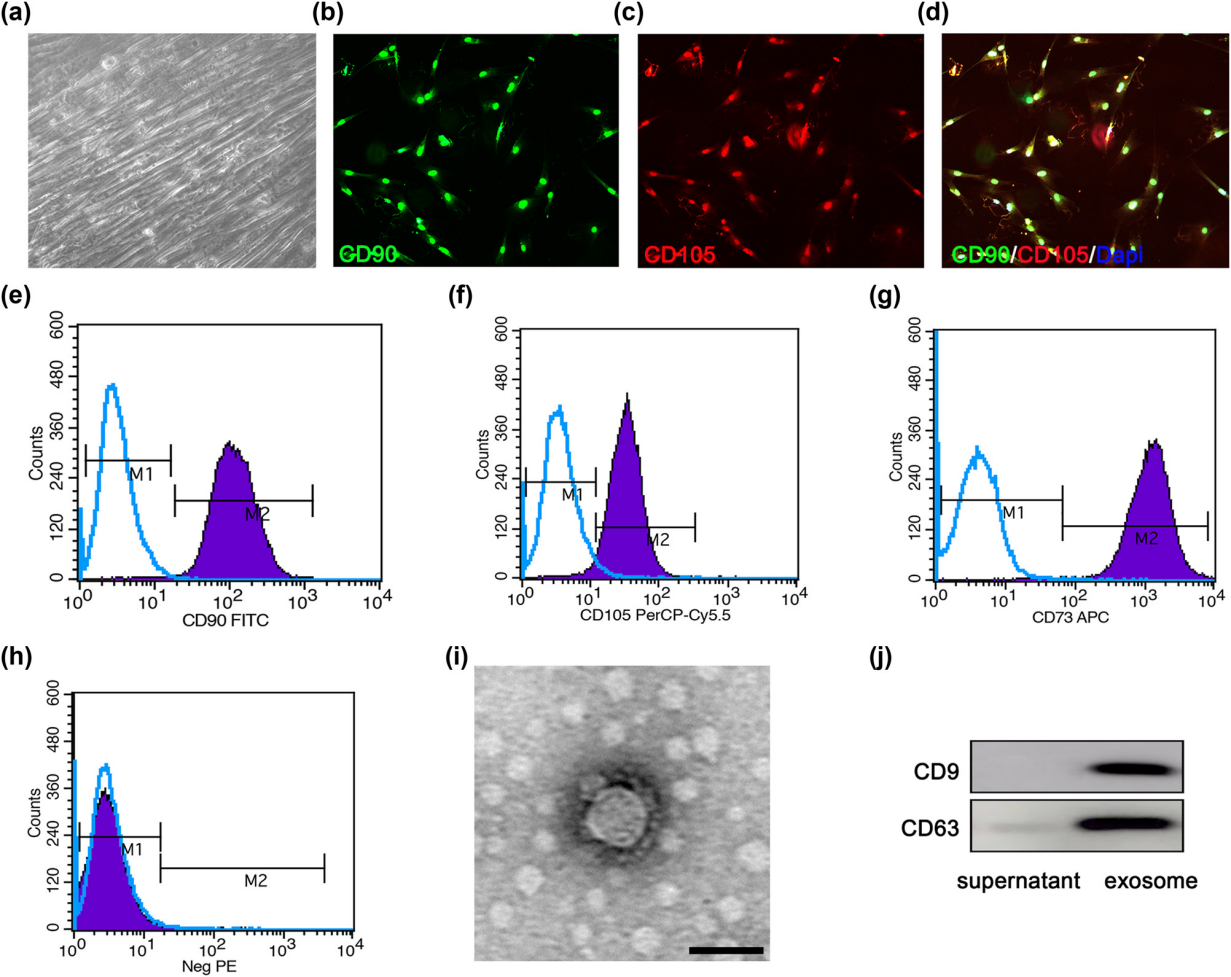
Identification of HUCMSCs and the HUCMSCs-derived exosomes. (a) Representative cell morphology of 4th generation HUCMSCs in phase contrast microscopy. (b–d) Double immunostaining with an anti-CD90 antibody (green) and an anti-CD105 antibody (red) to identify HUCMSCs. (e–h) Expression of CD90 (e), CD105 (f), CD73 (g), and negative molecules (Neg PE) (CD45, CD116, CD19, and HLA-DR) (h) on HUCMSCs detected by flow cytometry. (i) Representative transmission electron microscope image of the exosomes. (j) Exosomes surface marker protein expression of exosomes by Western blot. Scale bars = 50 µm in panels (a–d), 100 nm in panel (i).

The phase-contrast microscopy images showed that the 4th generation HUCMSCs were fusiform, and the cells were closely connected to form a spiral arrangement (Figure 1a). The biomarkers CD90 and CD105 of HUCMSCs were detected under fluorescence microscopy (Figure 1b–d). The results of flow cytometry indicated that HUCMSCs isolated and cultured in our study expressed MSC-specific marker proteins CD90, CD105, and CD73, and the expression rates were all >95%, suggesting that HUCMSCs were successfully isolated and cultured *in vitro*, and the purity met the experimental requirements (Figure 1e–h).

### 3.2 Characterization of exosomes derived from HUCMSCs

Transmission electron microscopy showed that the exosomes were round or elliptical membranous vesicles with significant heterogeneity, ranging from 30 to 200 nm in diameter. The membranous structure was visible in the periphery of the exosome vesicles and contained low-density substances (Figure 1i). The western blot results showed that the exosomes derived from HUCMSCs exhibited high CD9 and CD63 expression (Figure 1j).

### 3.3 Intravenous infusion of the HUCMSCs-derived exosomes significantly ameliorated the sensorimotor function and spatial learning in rats after TBI

**Figure 2:**
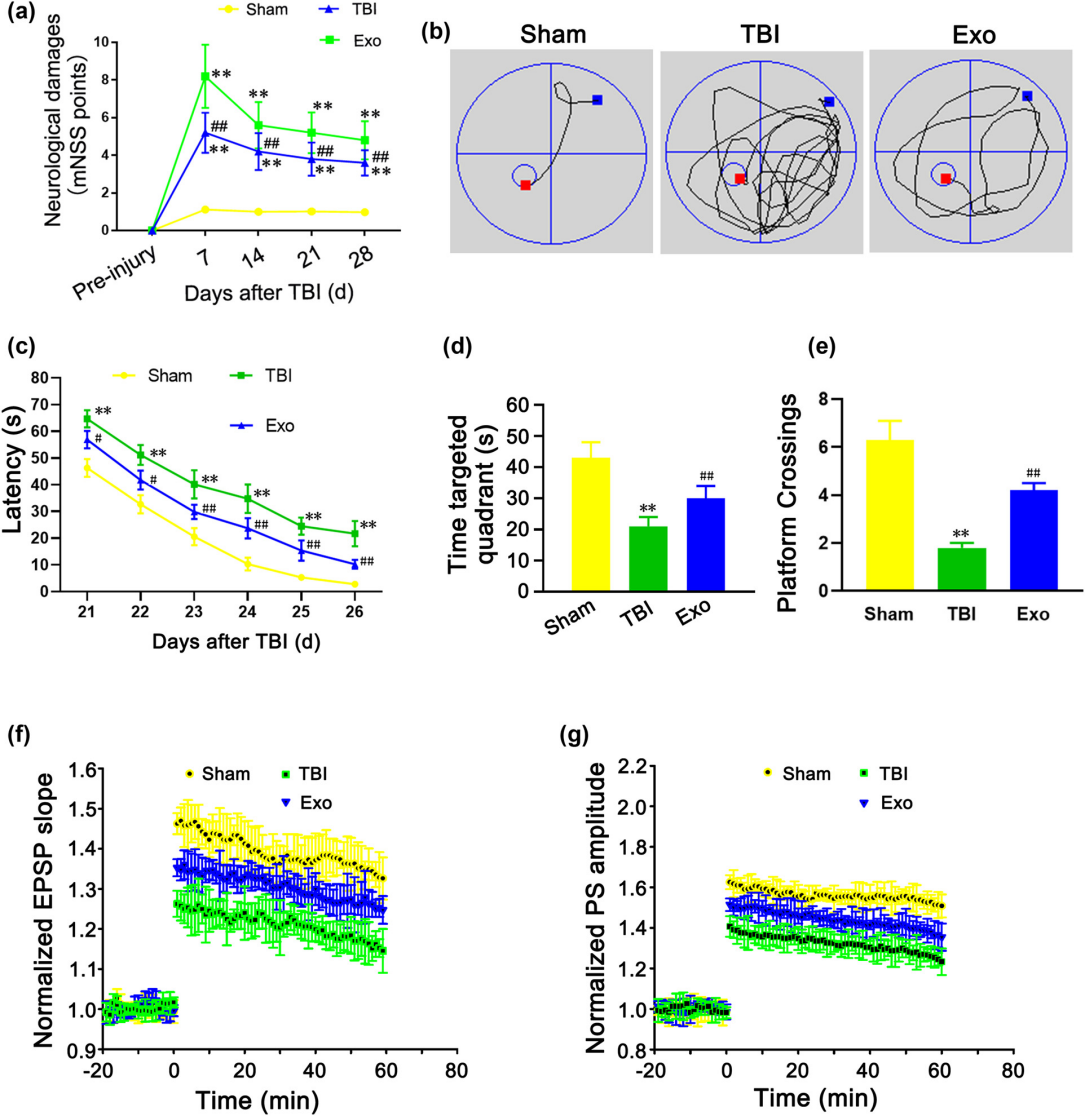
Administration of the HUCMSCs-derived exosomes markedly ameliorates sensorimotor function and spatial learning in rats after TBI. (a) Modified neurological severity (mNSS) score at 7, 14, 21, and 28 days after TBI. (b) Typical swim tracks in three groups. (c) The latency from 21 to 26 days after TBI. (d) The time spent in the targeted quadrant at 28 days after TBI. (e) The number of platform crossings. (f) Normalized excitatory postsynaptic potential (EPSP) slope at 28 days after TBI. (g) Population spike (PS) amplitude at 28 days after TBI. **P < 0.01 vs Sham group. ^#^ *P* < 0.05 and ^##^ *P* < 0.01 vs TBI group.

At 7, 14, 21, and 28 days after TBI, the mNSS scores of the TBI group and the Exo group were significantly higher than those of the Sham group (*P* < 0.01), while the mNSS scores of the Exo group were improved compared to the TBI group (*P* < 0.01) (Figure 2a). These results indicated that the HUCMSCs-derived exosomes could promote the repair of neurological deficits in rats after TBI. During the spatial learning phase, the swimming tracks of three groups were detected to analyze their learning ability (Figure 2a). At 28 days after TBI, compared with the Sham group, the latency of the TBI group was significantly prolonged (*P* < 0.01), and the time spent in the targeted quadrant and the number of platform crossings were significantly reduced (*P* < 0.01) (Figure 2c–e). We found that the latency, the time spent in the targeted quadrant, and the number of platform crossings were all enhanced in the Exo group compared to the TBI group (*P* < 0.01) (Figure 2c–e). The results of the MWM test suggested that the HUCMSCs-derived exosomes could promote learning and memory recovery in TBI rats. Compared to the TBI group, the administration of the HUCMSCs-derived exosomes in the Exo group significantly improved the EPSP slope (Figure 2f) and PS amplitude (Figure 2g).

### 3.4 Administration of the HUCMSCs-derived exosomes significantly reduced proinflammatory cytokine expression by suppressing the NF-κB signaling pathway

**Figure 3:**
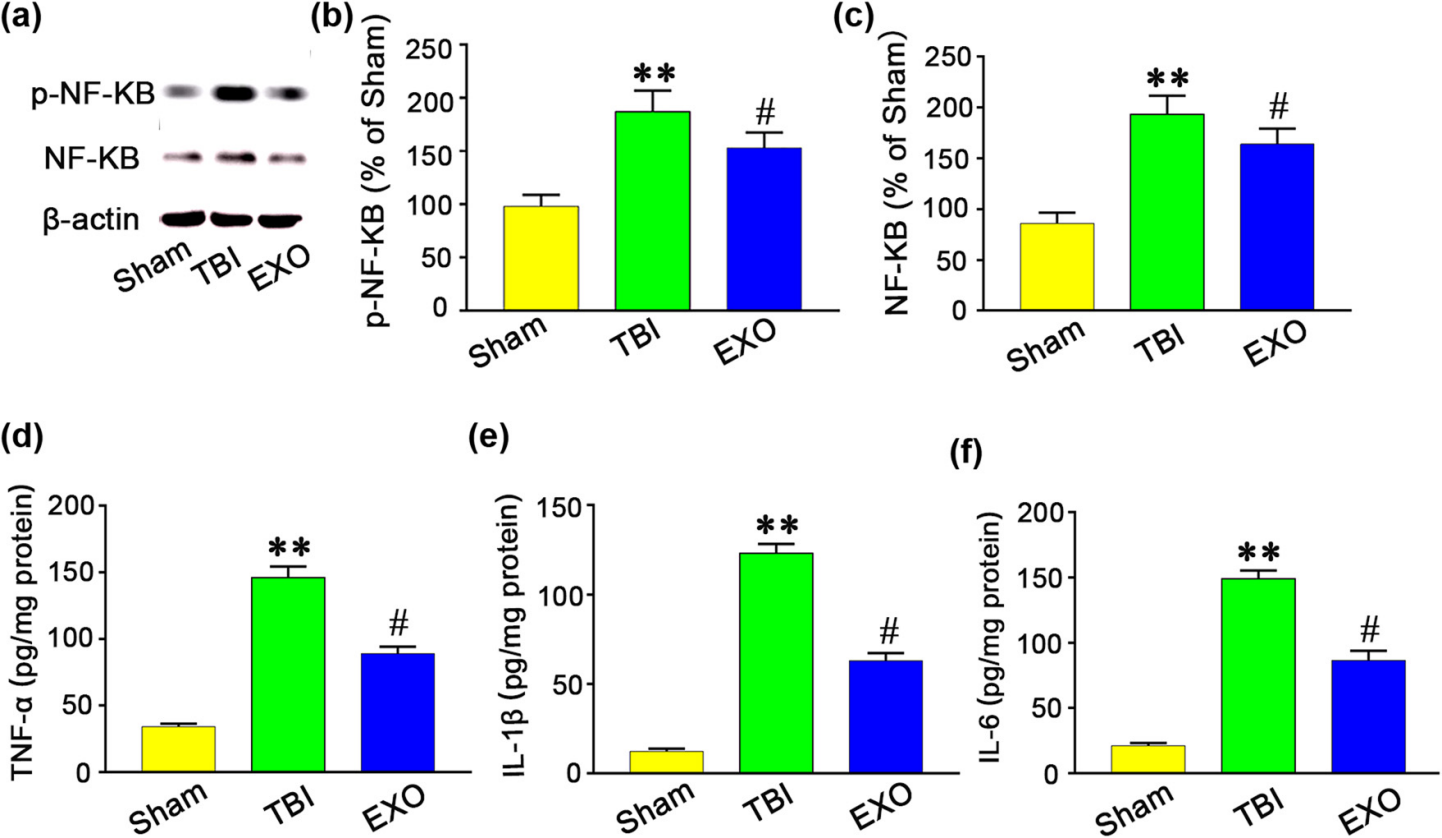
Administration of the HUCMSCs-derived exosomes reduced p-NF-κB and NF-κB and inflammatory cytokines (TNF-α, IL-1β, and IL-6) expression at 3 days after TBI. (a) Rats treated with HUMSCs-exo showed a decrease in NF-κB and p-NF-κB expression. (b and c) Western blotting showed that exosomes reduced p-NF-κB and NF-κB levels in the cortex. (d–f) TBI induced TNF-α, IL-1β, and IL-6 increase in rats. Rats treated with HUMSCs-exo showed a decrease in TNF-α, IL-1β, and IL-6 level. ELISA showed that HUMSCs-exo treatment reduced TNF-α, IL-1β, and IL-6 protein levels. **P < 0.01 vs Sham group. ^#^ *P* < 0.05 vs TBI group.

We further sought to assess whether the protective effect of exosomes could be attributed to inhibition of the NF-κB signaling pathway. At 3 days after TBI, the NF-κB expression was significantly increased in the TBI group compared with the Sham group (*P* < 0.01) (Figure 3a–c). However, the HUCMSCs-derived exosomes treatment significantly decreased NF-κB expression in the injured cortex compared with the TBI group (*P* < 0.05). We also found that TNF-α, IL-1β, and IL-6 levels were upregulated in the TBI group compared with the EXO group (Figure 3d–f). Quantitative analyses demonstrated that TNF-α, IL-1β, and IL-6 expressions in the Exo group were significantly reduced compared with TBI group (*P* < 0.05) (Figure 3d–f). The above findings suggest that behavioral recovery after administration of the HUCMSCs-derived exosomes may result from suppressing the NF-κB signaling pathway.

### 3.5 Administration of the HUCMSCs-derived exosomes attenuated neuronal apoptosis in the injured cortex of rats after TBI

**Figure 4:**
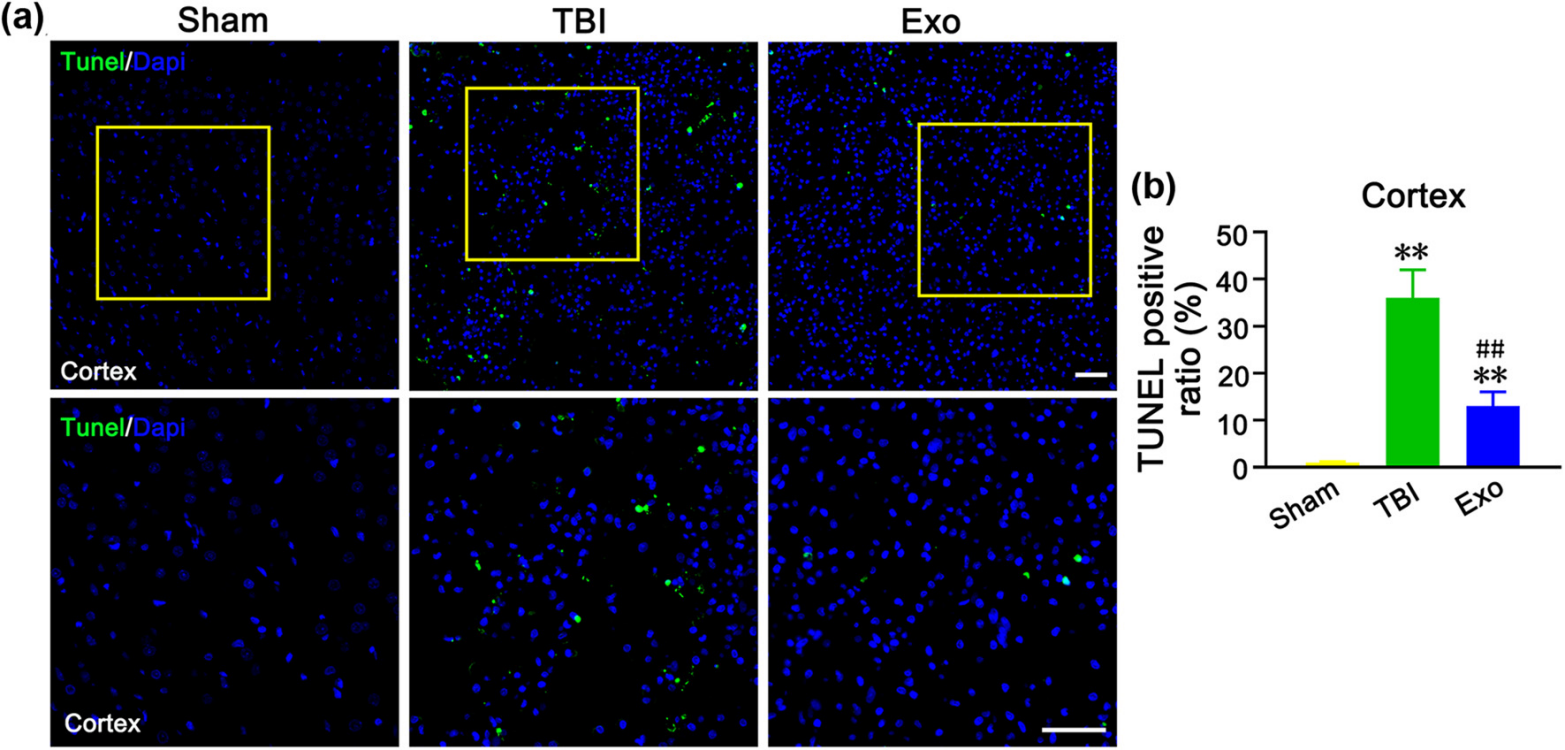
Administration of the HUCMSCs-derived exosomes attenuated neuronal apoptosis in the injured cortex of rats at 3 days after TBI. (a) TUNEL staining in the cortex in three groups. The image below is an amplified image of the yellow box in the image above. (b) Percentage of TUNEL positive rate in the injured cortex. **P < 0.01 vs Sham group. ^##^ *P* < 0.01 vs TBI group. Scale bars = 50 µm in (a).

To study whether the HUCMSCs-derived exosomes reduce neuronal apoptosis, rat brain tissue sections were subjected to TUNEL staining. We found the number of TUNEL positive cells at 3 days after TBI was significantly increased in the brain cortex of the TBI group compared to the Sham group (*P* < 0.01) (Figure 4a and b). We also observed that administration of the HUCMSCs-derived exosomes decreased the number of TUNEL positive cells compared to the TBI group (without any administration) (*P* < 0.01) (Figure 4a and b). These results revealed that the HUCMSCs-derived exosomes participated in alleviating apoptosis in the cerebral cortical neurons of TBI rats.

### 3.6 Administration of the HUCMSCs-derived exosomes significantly reduced injured cortex inflammation in rats after TBI

**Figure 5:**
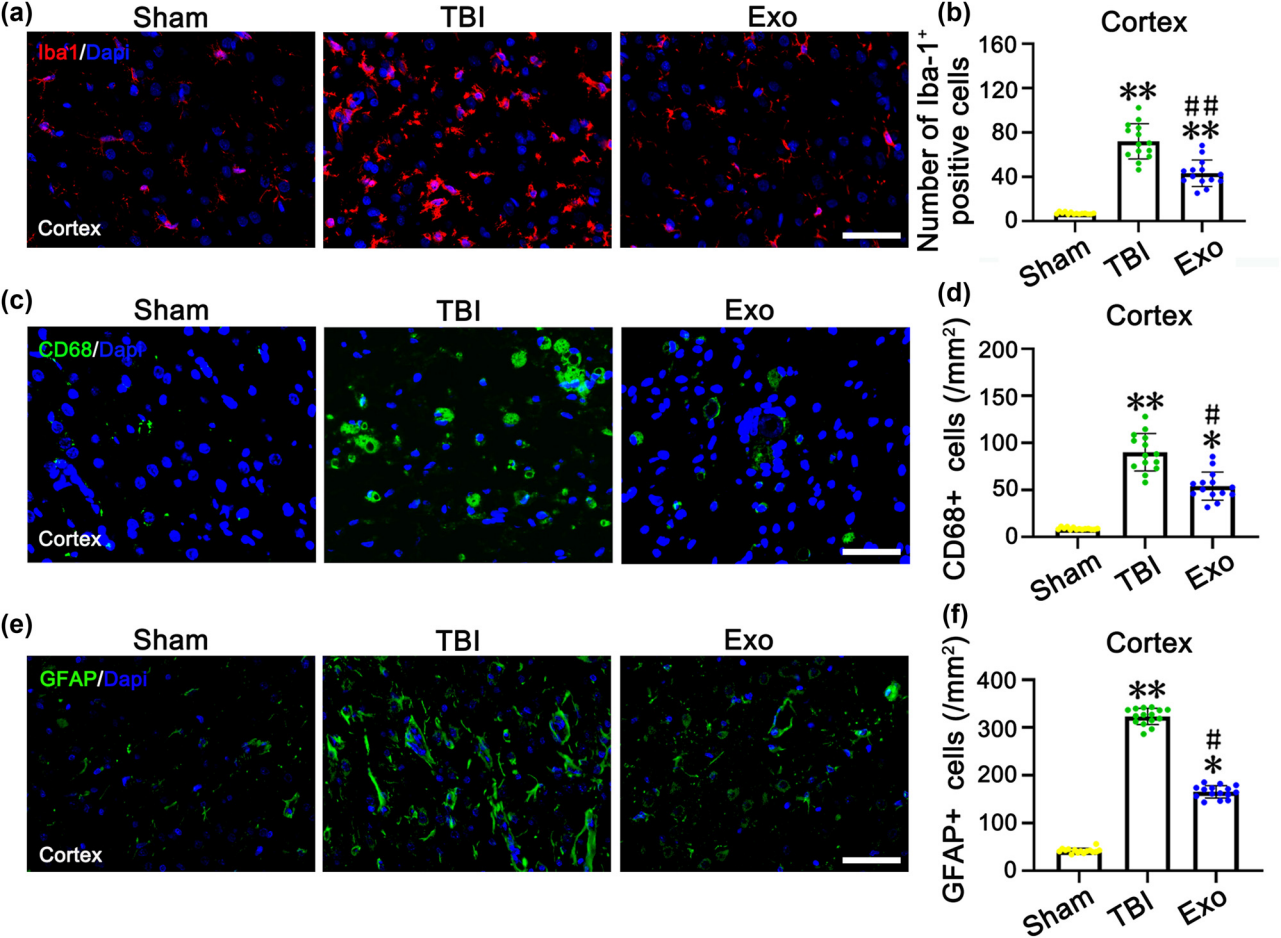
Administration of the HUCMSCs-derived exosomes obviously reduced the number of Iba1, CD68, and GFAP positive cells in the injured cortex of rats at 7 days after TBI. (a, c, and e) Iba1-positive cells (a), CD68-positive cells (c), and GFAP-positive cells (e) in the injured cortex of three groups. (b, d, and f) is the quantification of Iba1 (b), CD68 (d), and GFAP (f) positive cells in each group. *P < 0.05 and **P < 0.01 vs Sham group. ^#^ *P* < 0.05 and ^##^ *P* < 0.01 vs TBI group. Scale bars = 50 µm in panels (a, c, and e).

The number of Iba1-positive cells in the injured cortex was significantly reduced in the Exo group compared with the TBI group (*P* < 0.01) (Figure 5a and b). Administration of the HUCMSCs-derived exosomes significantly reduced the number of CD68 + cells in the injured cortex compared with the TBI group (without any administration) (*P* < 0.05) (Figure 5c and d). The number of GFAP + cells in the cortex in the injured cortex showed changes similar to those of the number of Iba1-positive cells and CD68 + cells after TBI. Moreover, the GFAP + astrocyte density in the injured cortex was significantly reduced in the Exo group compared with the TBI group (*P* < 0.05) (Figure 5e and f).

### 3.7 Administration of the HUCMSCs-derived exosomes significantly facilitated neuron regeneration in the injured cortex of rats after TBI

**Figure 6:**
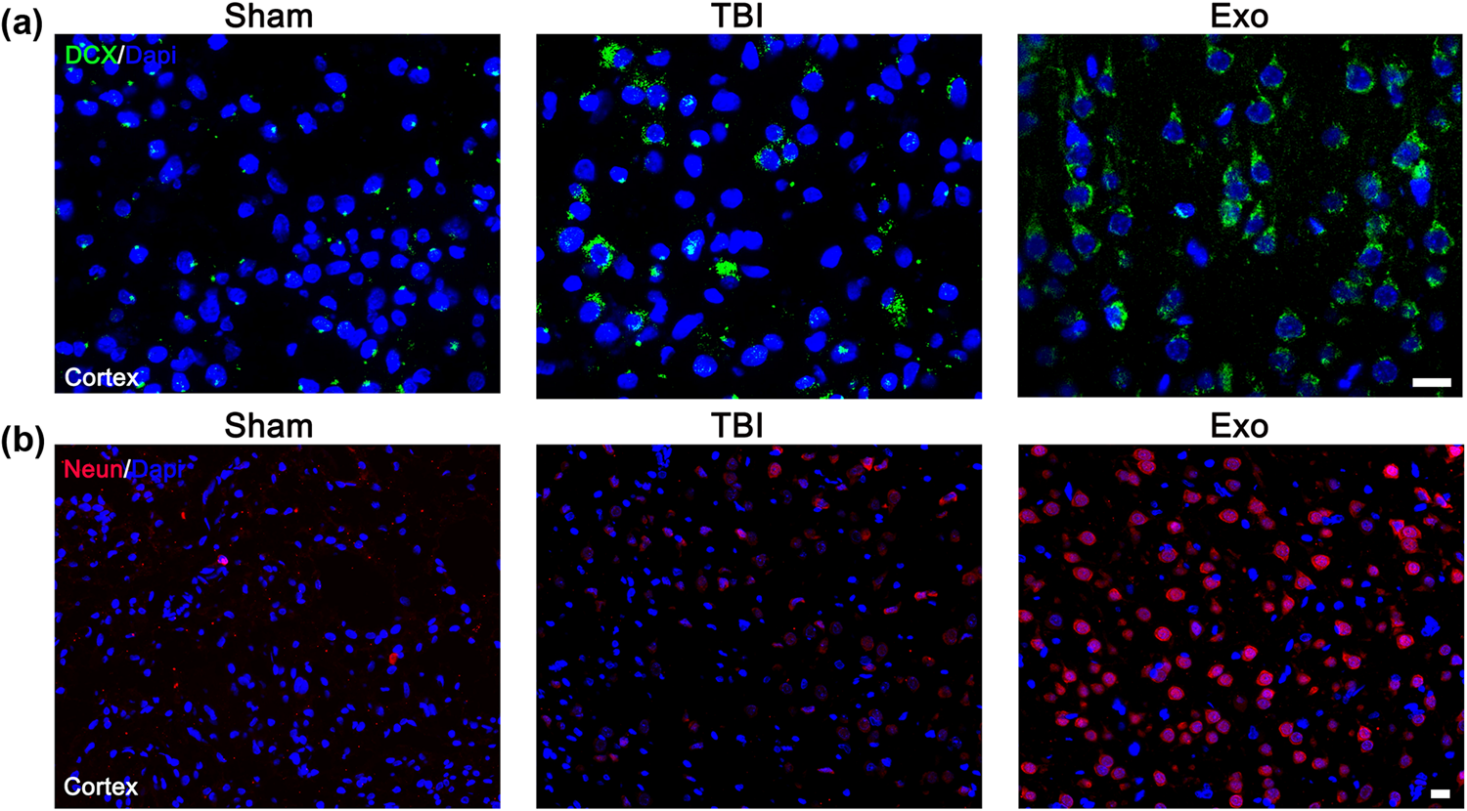
Administration of the HUCMSCs-derived exosomes obviously facilitated neuron regeneration in the injured cortex of rats after TBI. (a) DCX-positive cells in the injured cortex of three groups at 7 days after TBI. (b) NeuN-positive cells in the injured cortex of three groups at 28 days after TBI. Scale bars = 50 µm in panels (a and b).

DCX has been established as a marker for immature neurons, while NeuN is for mature neurons. Compared to the TBI group, the number of DCX-positive and NeuN-positive cells was significantly increased in the Exo group (Figure 6a and b). These results indicated that the administration of the HUCMSCs-derived exosomes could promote neuron regeneration.

## 4 Discussion

In the present study, we demonstrated that the HUCMSCs-derived exosomes had huge prospects for treating TBI in rat models. Significant improvements were observed in the HUCMSCs-exosome treatment group, including improved learning, memory and neurofunctional recovery, reduced glial scar formation, decreased neuron loss and cell apoptosis, and suppression of inflammation. Our study demonstrated that the HUCMSCs-derived exosomes could be a novel therapeutic approach for structural and functional TBI recovery.

Recent evidence suggests that the neuroprotective properties of MSCs can be attributed to the presence of multiple secretions of bioactive molecules that modulate the tissue microenvironment to repair and regenerate tissue [41]. Preclinical studies have substantiated the therapeutic effect of MSCs secretome in TBI. Soluble bioactive molecules and extracellular vesicles are various factors secreted by MSCs that induce neurogenesis, angiogenesis, neovascularization, and anti-inflammatory activity. Kim et al. demonstrated improved cognitive function using MSCs-derived CD63+ and CD81+ exosomes in a TBI mouse model [42]. Similarly, Doeppner et al. reported that the therapeutic effect of MSCs-derived exosomes was equivalent to MSC therapy in a rat model of stroke [43]. In our study, we indicated that intravenous infusion of the HUCMSCs-derived exosomes significantly ameliorated the sensorimotor function and spatial learning in rats after TBI by the mNSS scores and the MWM test. We found that administration of the HUCMSCs-derived exosomes decreased the number of TUNEL positive cells compared to the TBI group, which suggested that the administration of the HUCMSCs-derived exosomes attenuated neuronal apoptosis in the injured cortex of rats after TBI. Furthermore, the number of DCX-positive and NeuN-positive cells in the Exo group was significantly increased than that in the TBI group, which indicated that administration of the HUCMSCs-derived exosomes significantly facilitated neuron regeneration in the injured cortex of rats after TBI

Neuroinflammation plays a key role in the pathophysiology of central nervous system diseases such as cerebral ischemia/reperfusion [44] and TBI [45]. Microglial cells and astrocytes may also play an important role in neuroinflammation; microglia and astrocytes have been documented to be activated after brain injury and release an excessive amount of proinflammatory cytokines including TNF-α, and IL-6. Accumulation of these inflammatory factors increases the level of cell adhesion molecules and neutrophil infiltration. which is deleterious to neighboring cells, and enhances neural cell apoptosis, finally culminating in secondary brain injury [46,47]. Reactive astrocytosis is considered one of the pathological hallmarks of nerve injury [48]. Persistent reactive astrogliosis releases proinflammatory cytokines and exacerbates neuronal loss following inflammatory response [49]. Recent studies have shown that reactive astrocytes could be divided into “A1” or “A2” phenotypes based on different molecular markers [15]. Furthermore, a recent study found that microglial cells can generate IL-1α, TNF-α, and c1q, which induces A1 astrocytes activation, resulting in neuronal cell death [15]. However, NLY01, a GLP1R agonist, exerts a neuroprotective effect in Parkinson’s disease by inhibiting the formation of A1 reactive astrocytes [50]. Accordingly, the attenuation of activation astrocytes may be an important therapeutic strategy for CNS diseases. Some studies demonstrated that inhibition of the NF-κB signaling pathway was responsible for the neuroprotective effect after focal cerebral I/R injury [51]. Previous studies suggested that ADSCs-exosomes significantly limited the activation of the NF-kB signaling pathway in LPS-induced BV2 cells and suppressed the production of inflammatory cytokines, protecting neural cells from injury [52]. In our study, the number of Iba1-positive cells and CD68 + cells in the injured cortex was significantly reduced in the Exo group compared with the TBI group, which demonstrated that administration of the HUCMSCs-derived exosomes significantly reduced injured cortex inflammation in rats after TBI. Moreover, the GFAP + astrocyte density in the injured cortex of the Exo group was significantly reduced than that of the TBI group. Our study showed that the HUCMSCs-derived exosomes suppressed the activation of microglia and astrocytes and downregulated inflammatory cytokine expression by decreasing the activation of the NF-kB signaling pathway.

It is currently not possible to extract exosomes in large quantities and with high purity. Nonetheless, the present study demonstrated the potential of stem cell-derived exosomes in the treatment of TBI. Further studies are required to elucidate the mechanisms underlying stem cell-derived exosomes’ protective and regenerative effects.

Given the paucity of clinical data supporting the clinical application of exosomes therapy, the use of exosomes at the clinical level is still at the theoretical and experimental stages. Extensive progress made in the production of clinical grade exosomes will certainly enable the development of new therapeutic strategies to reduce the mortality rate and improve the quality of life of this particular patient population.
